# Addressing seeming paradoxes by embracing them: small state theory and the integration of migrants

**DOI:** 10.1186/s40878-021-00222-8

**Published:** 2021-04-20

**Authors:** Thomas Kolnberger, Harlan Koff

**Affiliations:** 1 Faculty of Humanities, Education and Social Sciences, Maison des Sciences Humaines, Université du Luxembourg, 11, Porte des Sciences, L-4366 Esch-Belval, Luxembourg; 2 INECOL, Carretera antigua a Coatepec 351, El Haya, Xalapa, 91073 Veracruz México; 3 Department of Politics and International Relations, University of Johannesburg, PO Box 524, Auckland Park, 2006 South Africa

**Keywords:** Exclusion, Integration, Luxembourg, Migration, Small state theory

## Abstract

This article examines the integration of migrants in Luxembourg within the framework of small state theory. Within the comparative scholarship on migration, small states are often presented as “success stories.” This research questions this assumption and empirical data presented here indicates that many contradictions exist within Luxembourg’s migrant integration model. The country’s “success” in fact does not reflect the levels of integration of migrants nationally as significant inequalities are present in Luxembourg. However, the analysis of Luxembourg presented here illustrates how small states have coherently embraced many paradoxes that are inherent to integration strategies throughout Europe with the goal of promoting peaceful coexistence.

## Introduction

Numerous seeming paradoxes characterize migrant integration politics. For example, nativist reactions are often strong in areas that are economically dependent on migrant labor (see Moldes-Anaya et al. 2019). Similarly, the securitization of migration policies often includes human rights language (Pajnik 2007). Observers of integration policies focusing on migrant women, such as Anthias et al. (2012), Espinosa (2018) and Sacchetti (2016) contend that a strength of these policies is their ability to respond to differentiated needs but simultaneously, a weakness is differential understanding of integration by different users which contributes to ambiguity and mixed results. Even the notion of migrant integration itself presents paradoxes. Whereas previous discussions of integration focused on improving migrant participation in host societies (see Koff 2008), more recent discussions have contended that traditional understandings of integration reflect outdated “neocolonial forms of knowledge” (Schinkel 2018) and “methodological nationalism” (Favell 2019) that reinforce hierarchical orders. Malik (2015) has contended that migrant integration strategies were viewed as answers to social problems in Europe, whereas now they are often perceived to have been the cause of them. These debates highlight a significant problem with the notion of migrant integration. The more that such strategies are perceived to be necessary, the more they are viewed as failures. When integration is supposedly successful, then policies can be criticized for being unnecessary. For this reason, integration strategies are caught between a rock and a hard place in European political agendas.

This article examines these integration paradoxes through the lens of small state theory. In doing so, it questions whether this theory which has been used to present small states as effective and efficient political and economic actors can be applied to migrant integration. In fact, small states are often viewed as successful examples of integration. Observers often point to perceived lower intensity of overt racism, increased levels of economic integration and the extension of political rights, such as local voting and access to welfare (see Fetzer 2011). This article asks: Can small state theory address seeming paradoxes in migrant integration, and in doing so, can it contribute to migrant integration theory? In responding to this question, the article aims to highlight specific small state governance characteristics that either contribute to or detract from successful long-term migrant integration. It is divided into four parts. Following this introduction, part two presents small state theory as the conceptual approach for analysis. Part three introduces a conceptual “small-state” model for migrant integration which is then empirically applied to Luxembourg. Finally, part four presents the article’s conclusions.

## Small state theory and the embrace of paradoxes

Like debates over migrant integration, discussions on small states in global affairs are often characterized by numerous paradoxes. On one hand, early works highlighted small state vulnerability in military and economic security terms as scholars argued that small states needed to join alliances to survive, both politically and economically (Keohane 1969; Handel 1981). In response, other observers, such as Katzenstein (1985, 2003) and Cooper and Shaw (2009) highlighted distinct approaches to policy-making which promoted innovation and prosperity amongst small states. Small states are portrayed as both vulnerable and adept at risk governance (see Koff and Maganda 2015; Koff et al. 2020; Lusa 2019). Another seeming paradox is the recognized commitment of small states to international norms in constructivist perspectives (see Siitonen 2017; Graham and Graham 2019; Nadalutti 2020) whereas neo-functionalist views describe small states as global actors driven by economic logic and incentives for trade optimization (see Schiff 2014).

Katzenstein (2003) has indicated the following six defining characteristics of small state theory: (1) small states have relatively homogeneous populations; (2) small states are open to international economies; (3) small states create niches in global economies; (4) small states promote social solidarity due to perceived notions of vulnerability to external shocks; (5) small states amplify their influence through regions; and (6) small states possess efficient and effective governments because of the their propensity for interpersonal relations. These characteristics are meant to explain why small states are influential in global affairs and stable domestically. However, comparative, cross-regional research has also shown how these very characteristics have contributed to governance challenges in transnational policy sectors. For example, Koff and Maganda (2015) have indicated that these characteristics can be counter-productive in transboundary water management. Their research shows how openness to international economies actually affects the homogeneity of small state populations by exacerbating inequalities; niches in global economies contribute to risk exposure; the propensity for social solidarity reinforces national resource management at the expense of transnational cooperation; and above all the propensity for interpersonal relation can prevent the evolution of legal/institutional responses to political issues, especially when they are transnational in nature.

The literature on small states, citizenship and borders similarly highlights these paradoxes. Small states are perceived to be cosmopolitan and globalized economically but provincial socially (see Clément 2013). In terms of integration governance, small states are often presented as committed to social welfare, but exclusionary in deciding who gets access to such programs (see Burlacu and O’Donoghue 2013). Finally, small states are often viewed a models for consensus-building and mediation but concertation often takes place behind closed doors, thus indicating a lack of transparency in decision-making (Katzenstein 1985).

The specific literature on small states and migrant integration can be divided into three different branches. The first group of studies focuses on migration within the context of the labor market needs of small states. It discusses economic opportunities, economic regulation, welfare, and corporatist decision-making. The first characteristic of this literature is its recognition of differences between transnational labor flows in large and small states. Whereas most of the traditional literature on migration discusses this phenomenon within the context of unskilled labor, security, protectionism and nativist reactions to immigration (see Lazaridis and Wadia 2015; Koff 2017), the literature on migration to small states recognizes more complex needs and nuanced reactions. To begin with, small states are characterized by mixed flows of migrants that include high percentages of skilled labor on which these countries depend. This has been empirically shown in countries such as Luxembourg (Fetzer 2011), Singapore (Yeoh and Lin 2013), Botswana (Campbell 2006), and Qatar (Fargues and Shah 2017), to name a few. These countries are characterized by bifurcated labor markets in which low skilled labor is recruited from neighboring countries in the form of cross-border workers (see Burlacu and O’Donoghue 2013) whereas high-skilled migration, especially that linked to the financial sector, comes from other advanced industrial states such as EU member countries and the United States. Within this literature, small states are often applauded for economic planning, welfare management and long-term migrant economic integration (Nannestad 2007).

The second branch of the literature on migration and small states reinforces the seeming success of these countries in managing migration. Specifically, this scholarship recognizes that small states are characterized by migration and citizenship debates that do not reflect those present in large advanced industrial countries (see Thompson 2014). On one hand, scholars such as Lee (2016) have presented small states as models of “cosmopolitan citizenship.” These studies document the transnational nature of citizenship in small states often relating this phenomenon to the notion of “global cities” (see Sassen 2001). Through labor mobility and transnational economic networks, these countries are often regarded as the embodiment of globalization.

The third branch of the integration literature on small states introduces the seeming paradoxes presented above. Whereas political and economic integration are perceived successes in these countries, social integration is generally viewed as lagging, thus uncovering the existence of marked social hierarchies. Specifically, geographic studies of socio-spatial inequalities have shown that immigrants are not as well-integrated as the literature on political citizenship would indicate because residential segregation exists in small states. Nagy (2006) conducted an ethnographic study of residents in urban Qatar and demonstrated how residential segregation and official political discourse mutually reinforced “ideologies of difference” between citizens and foreigners in Qatari cities. In Luxembourg, which represents a very different model of governance and socio-spatial planning, scholars such as Lord et al. (2014) have examined the interaction between migration and socio-spatial structures and discussed how these processes have contributed to increased gentrification, segregation and the diffusion of different social groups in the Greater Region as a result of increasing social disparities.

This body of integration scholarship highlights an important small state paradox. It focuses on the co-existence of transnational labor markets and cosmopolitan citizenship on one hand, and domestic social control/hierarchies on the other. This co-existence of inclusion and exclusion indicates that small states, such as Luxembourg, should not necessarily be presented as absolute successes in terms of integration strategies. Therefore, this article addresses small state theory within the framework of migrant integration debates in the European Union by examining the paradoxes that it seems to embrace.

## Migrant integration in Luxembourg: an examination of small-state theory in practice

As stated above, the literature on migration often focuses on how well small states integrate migrants. This article contends that this is not necessarily the case. It does so through an examination of migrant integration in Luxembourg which can be considered representative of “small states” in various ways. While the definition of the small state is elusive, state size matters (Maass 2009; Wolf 2016): In January 2020, Luxembourg had a population of 626,000, distributed over an area of 2586.4 km^2^ (global rank in population and size: 164 and 167 respectively out of 194). It has an economy that is based on consensus-building and corporatist governance (see Clément 2013) and its leadership has effectively established a niche economy in banking and finance (Walther et al. 2011). Economic planning occurs through corporatist structures such as the *Tripartite* which brings together representatives of government, business and labor unions. Luxembourg is also deeply embedded in regional integration at the macro and micro levels as a founding member of the Europe Union and one of its recognized capitals hosting among others the European Court of Justice, European Court of Auditors and European Publication Office. It is the economic and political center of “The Greater Region” as well. This cross-border polity includes the sub-national regions of Lorraine (France), Wallonie (Belgium), the German-speaking Community of Belgium, Rhineland-Palatinate (Germany) and Saarland (Germany) (see Evrard 2016). The area covers 65,401 km^2^, with a population of approximately 11.2 million inhabitants. Most importantly, almost 200,000 commuters cross the borders into Luxembourg on a daily basis as the Greater Region provides the country with an important source of labor, especially in the service sector (see Thomas 2016).

Luxembourg can also be considered a critical case through which to study immigration politics in small states. It has a significant migration history as a sending state in the eighteenth and nineteenth centuries. The country’s industrialization, beginning in the second half of the nineteenth century, led to large-scale labor migration, first from Italy, Germany and then from Portugal. The economic transformation which occurred in the 1980s and 1990s from an industrial state to a global financial and regional political capital diversified migration as the country is host to high-skilled migrants from all over the world. Consequently, unlike other small states, such as Botswana or Qatar, migration is not a new phenomenon in Luxembourg. Statistically, over 40% of the residents in Luxembourg are non-nationals (see Kmec and De Jonge 2019).

Research for this article is based on mixed methods. In November 2004, the Council of the European Union agreed on common basic principles for immigrant integration policy (CBP) in the European Union (European Union 2004). In April 2011, the Prime minister’s office “le Gouvernement en Conseil” of Luxembourg assigned the national CES (Conseil économique et social), a permanent advisory body nominated by the government, to evaluate the “National action plan for integration and fight against discrimination” (MFI 2010), which is the individual Luxembourgish “Roadmap” to fulfill the Council’s request. The Council’s suggestions also foresee periodic evaluation of the CBPs. The CES called the University of Luxembourg for support to realize this evaluation and to draft a report “Evaluationsbericht” in cooperation with the CES (T. Kolnberger and C. Baltes-Löhr with M. Menei, Attachée de direction 1^er^ en rang, and M. Nati-Stoffel, Secrétaire général of the CES, unpublished *Evaluationsbericht* *zum* *"Plan d'action national pluriannuel d'intégration et de lutte contre les discriminations 2010–2014"* - *interner Gebrauch*). This preliminary report is unpublished (released for academic research by Gary Kneip, President of the CES, 2013–2015) and became the basis for the official “Avis” (advisory report or expert’s opinion, see: CES 2014). These reports inform the present article.

The empirical data consists of three parts: one entirely qualitative, one conducted through mixed methods research (interviews and policy analysis) and a quality control mechanism. The first part comprised 27 semi-structured in-depth interviews with three groups of stakeholders: (a) senior officials and top echelons of 17 ministries and other government organizations (National Job center and labor office ADEM, https://adem.public.lu/fr.html; the National Immigration Welcome Service OLAI, http://www.olai.public.lu/en/index.html, etc.); (b) the so called “social partners” (“partenaires sociaux”), i.e. three labor unions, Council for Foreigners CNE, the Association of Luxemburgish towns and communities SYVICOL or the Employers’ Association UEL, and (c) representatives of the “civil society”, which are fully or partly state-sponsored “non-government” organizations (“associations conventionnées”).

The interviews were recorded and prepared for computer-assisted qualitative data analyses using MAXQDA software. Part two (mixed methods) consisted of two questionnaires (Fiche évaluation des politiques d’intégration and Fiche bilan 2011–2012) submitted to the ministries. However, the response rate of this initiative was not as high as the research team had hoped: only eight of 14 offices returned questionnaire one and only four returned questionnaire two. Regrettably, the Ministry of Foreign and European Affairs, which also comprises the Directorate of Immigration (including notably the Department for foreigners, the Department for refugees and the Department for returns) refused to participate with the justification that they were “not responsible for integration or the CBPs” (telephone request for interview, September 2013 by CES). Even though this lower response rate represents a quantitative limitation of the study, the 12 questionnaires returned did provide interesting qualitative information. These responses were examined in comparison with interview responses and reviews of key policy documents that guide Luxembourg’s migrant integration strategies, permitting a triangularization of sources despite the lower response rate. Nonetheless, due to this limitation of survey research, the empirical findings below will only reference data generated through the interviews and policy review.

Finally, the research team publicly presented empirical results as a quality control mechanism. The study was presented and discussed with all national migrant integration NGOs and public stakeholders (roundtable discussion). Additionally, a feedback-round with further civil servants of the ministries involved was organized before the presentation of the report.

In review, the theory and methodology of this three-stage analytic strategy was informed by advantages and shortcomings of thematic and narrative interpretations. The two approaches work together when a “narrative analysis”-step approach investigates and brings forward broad aspects of research (the qualitative part of the interviews), followed by thematic in-depth analyses (the qualitative part of the questionnaires). Narratives about the catchword “integration” proved to be very useful for identifying commonalities amongst participants, through the idea of shared themes. The questions (for the semi-structured investigative) interview, which we prepared as a default-option, turned out to be superfluous because after the general introduction the interviewees proceeded immediately to what we called “self-thematization”, meaning the independent choice of their substantive focus und thematic prioritization. We conclude that thematic and narrative methods, when integrated, produce a multidimensional understanding of the perception and reality of “integration” (compare McAllum et al. 2019; Kohler Riessman 2008).

Reviews of policy and academic literatures as well as official databases complemented this research. The authors also consulted the Migrant Integration Policy Index (MIPEX).

## Migrant integration and stakeholder perceptions

The interviews conducted for this study represent an interesting starting point for this analysis on small state theory and integration because stakeholders confirm many of the paradoxes present in Luxembourg’s migration debates. When asked about the differentiation between humanitarian migration (i.e. asylum seekers) and economic migrants, the generalized response from government representatives was “We have to fulfill our humanitarian obligation” (personal interviews), thus downplaying responsibilities toward economic migrants. In fact, the responses from the interviews generally seemed to contradict the view of small states as “integration success stories.” When asked to reference any “positive examples” of migrant integration, only 12 of the 27 interviewees would do so.

Similar views were expressed during a series of questions related to social cohesion and migration. When asked if migration is a threat to social cohesion in Luxembourg, 13 interviewees responded “yes,” zero said “no” and 14 chose not to respond. The next question asked “Is there a threat to social cohesion in Luxembourg due to the growing ‘cultural distance’ of the new migrant groups?” Fifteen officials agreed with the notion of an increased threat to cohesion, three disagreed and nine did not respond. Collectively, these responses are significant because they indicate that government officials who work in integration affairs do not necessarily view integration positively. Moreover, the high rate of non-responses can be interpreted as indifference or skepticism which officials do not wish to express. The fact that there is no overwhelming expression of support for integration dominates the responses to these questions.

The economic bases of integration in Luxembourg were also highlighted in the interviews. When asked “Is migration a threat to social cohesion in Luxembourg due to ‘crumbling’ abilities of the state to guarantee social transfer payments in the future?” 22 respondents answered affirmatively, one answered negatively and four did not respond. This was followed by the question “Is the gap between rich and poor widening and a threat to social cohesion?” to which 21 said “yes,” zero said “no” and six did not respond. Finally, respondents were asked “Is there spreading/increasing xenophobia in Luxembourg?” Sixteen respondents agreed, zero disagreed and 11 chose not to answer. Collectively, these answers illustrate how respondents view migration as a “threat” to social cohesion in Luxembourg, and consequently they seem to show that “societal security,” defined as the maintenance of social stability, is as much an issue in the country, as it is in large advanced industrial states, seemingly contradicting the literature on small states and migration. Moreover, these responses reflect the paradoxes described above in which small states promote migration for economic well-being but social integration remains a challenge.

Luxembourg’s political landscape also reflects these paradoxes. In general, nativism is largely absent from Luxembourgish migration discussions and discrimination is openly condemned. For example, Luxembourg has not witnessed a rise of the far-right in its political system, which is often considered an indicator of anti-immigrant sentiment. Nonetheless, local experts, such as Fernand Fehlen have argued that far-right movements are rejected less for identity-based arguments and more in the name of socio-economic stability. Fehlen contends that as long as Luxembourg is in a good economic position, the far-right will remain on the margins (Ziemetz 2018). In fact, during the 2019 European Parliament elections the conservative-populist Alternative Democratic Reform Party (ADR), which holds four of the 66 seats in the Luxembourgish Chamber of Deputies, aligned itself with the anti-immigrant Wee2050 - nee2015 citizens movement (https://nee2015.lu). The party only won 10% of the vote and no seats in the European Parliament in contrast to the success of far-right parties in these elections continentally (Rankin 2019).

While these results indicate a lack of large-scale political nativism in the country, this does not necessarily signify widespread support for immigrants politically. This lesson was learned by Prime Minister Xavier Bettel in 2015 when he introduced a constitutional referendum which asked whether foreign nationals should be given the right to vote in parliamentary elections. The referendum was defeated overwhelmingly by 78% of the vote (Forum 2015). Consequently, these trends confirm the seeming contradictory opinions expressed by many public officials in the interviews. These perspectives already reflect an integration reality that seems more complex than generally indicated in the literature.

## Migrant integration in Luxembourg: an analysis based on small-state theory

**Fig. 1:**
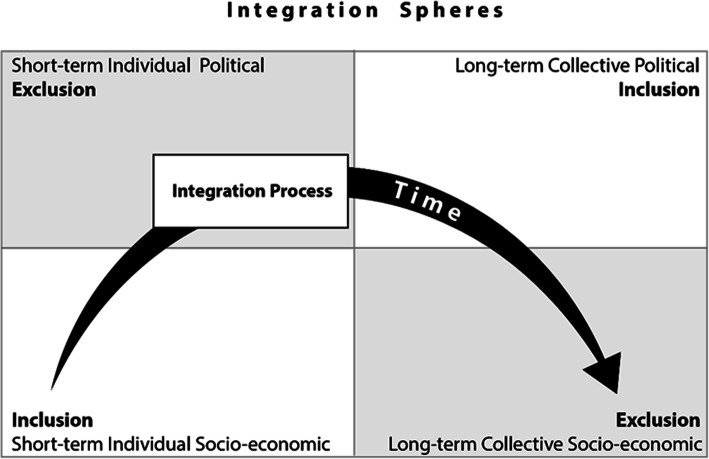
The Co-existence of Inclusion and Exclusion in Luxembourg’s Migration Regime

The established literature on small state governance (see Katzenstein 1985; Ingebriten 2010; Koff et al. 2020) has underlined the propensity of small states to promote social stability due to perceived vulnerability to external shocks, such as migration. They do so by framing migration pragmatically and pursuing a system of differentiated integration (Burlacu and O’Donoghue 2013). In her work on cross-border conflict over water resources, Kauffer (2014 and 2017) contends that cooperation and conflict should not be viewed as competing positions. Instead, she argues that they are intersected in political relations and they co-exist along a continuum in transnational debates. This logic is present in migrant integration strategies in Luxembourg where inclusion and exclusion co-exist at the individual and collective levels and between short-term and long-term strategies. On one hand, individual migrants, especially non-Europeans (also known as third-country citizens) are faced with short-term formal economic barriers which are utilized by the Luxembourgish state to regulate labor markets (these measures largely follow EU norms). Nonetheless, due to the presence of economic activities and a lack of overt discrimination, indivual migrants generally negotiate long-term economic integration in the country. This lack of overt discrimination has also contributed to collective short-term social integration for migrant groups in the country. For example, unlike other European states, Syrian asylum-seekers have generally been welcomed in Luxembourg and provided accommodation and services without visible negative reactions. On the contrary, long-term socio-economic integration has been hindered by segmented labor markets where language is often a barrier to integration. The combination of segmented labor with high housing prices has led to spatial exclusion and socio-economic hierarchies. As mentioned earlier, formal barriers to migrant political integration also exist. Consequently, integration and exclusion formally exist within the Luxembourgish migrant integration model in both short-term and long-term strategies. This is illustrated in Fig. 1 which shows how individual integration co-exists with collective exclusion.

This situation, however, maintains peaceful coexistence within the country. Citizens accept the legitimacy of migration because they are economically privileged by it and political integration remains dependent on citizenship. Conversely, migrants accept their collective secondary status because they individually benefit economically in the long-term once they overcome short-term market barriers.

These trends indicate that small state theory seems to apply to migrant integration by embracing the integration paradoxes identified in the migrant integration literature. Instead of illustrating absolute “successful integration” as indicated in recent small state scholarship, small states seem to actively promote many of the paradoxes indicated above with the goal of fostering peaceful coexistence. Migrants and citizens share certain privileges but differentiation is present as well. In terms of policy logics, there seems to be a gap between normative commitments to asylum seekers resulting from Luxembourg’s dedication to human rights in foreign policy and utilitarian management of economic migrant integration. These tendencies are consistent with small state theory. They are empirically examined in detail below.

### The co-existence of integration and exclusion in Luxembourg: embracing integration paradoxes

The coexistence of integration and exclusion of migrants in Luxembourg was highlighted by public officials interviewed for this project. On one hand, many officials agreed with supporting the economic claims of migrants. Conversely, many interviewees contended that full citizenship and related rights (particularly the right to vote on the national level) should be restricted. A “legal discrimination” between citizens and non-citizens was viewed as morally acceptable. Similarly, public officials viewed the segmented labor market in Luxembourg as “natural,” contending that public sector employment should be reserved for Luxembourgish citizens (with a few exceptions, proficiency in the three official languages, i.e., Luxembourgish, French, and German, is the precondition for public service). These opinions reflect migration dynamics in Luxembourg.

These trends are evident in the comparative research on migration in Luxembourg. As a starting point, this research project consulted the Migrant Integration Policy Index (MIPEX). This index measures policies to integrate migrants in all EU Member States, Australia, Canada, Iceland, Japan, South Korea, New Zealand, Norway, Switzerland, Turkey and the USA. It has developed 167 indicators to create a multi-dimensional instrument that evaluates and compares what governments are doing to promote the integration of migrants.

The index is interesting because it presents data that does not necessarily confirm Luxembourg’s place as a “migration success story.” In fact, Luxembourg is only ranked 15th out of 38 states with an overall integration score of 57 out of 100 (http://www.mipex.eu/key-findings), behind countries with conflictive migration debates such as Italy, Spain and the United States. More specifically, individual scores seem to verify the coexistence of inclusion and exclusion as indicated by the model presented above. Luxembourg’s highest scores are found in political categories, such as “Family Reunion,” (68/100), “Permanent Residence,” (64/100), “Political Participation” (81/100) and “Access to Nationality” (68/100). The lowest national scores are in “Labor Mobility” (42/100), “Health” (48/100) and “Education” (43/100).

These trends are interesting because they do not necessarily reflect public debate on migration in Luxembourg. Most of the recent international attention on migration to Luxembourg has focused on the referendum on national voting rights and access to citizenship. The restrictions in these fields seem to highlight political exclusion but this is not really an issue in the country. In fact, even holding a referendum on national voting rights can be considered an indicator of political inclusion because this is a very evolved debate. Conversely, public migration discourses generally underline Luxembourg’s economic dependence on migrant labor as an indicator of economic integration. Instead, segmented labor markets have established clear economic hierarchies and inequality is a structural characteristic of Luxembourg’s integration model. Only 25% of the country’s workforce is Luxembourgish. The large public sector, which employed 40,007 mostly Luxembourgish citizens in 2017, is paid for by the private work sector which employs 336,039 migrants, including 116,328 EU citizens, 17,886 non-EU citizens and 201,825 cross-border workers (Statec 2019).

The qualitative comments in MIPEX’s Luxembourg country page explain these tendencies more clearly. MIPEX concludes that “integration is the reality for the mostly long-settled immigrants in this small, wealthy, multilingual country” (http://www.mipex.eu/luxembourg). The report indicates that more than half of Luxembourg’s EU and non-EU migrants are university-educated, with high rates of political participation. Also, Luxembourg actively promotes political participation through considerable funding of migrant non-governmental organizations and structural financial support by the Ministry of Family and Integration of the *Comité de liaison des associations issues de l’immigration*, a platform of more than 200 migrant NGOs (https://www.clae.lu/). Conversely, MIPEX discusses socio-economic exclusion in the following way: “Inequalities do appear in terms of the social concentration of immigrant pupils in disadvantaged schools, the under-representation of immigrants and their descendants in the public sector (the greatest of any Western country), gaps in income, in-work poverty, poverty-risk and uptake of training, especially for non-EU citizens, experiences of discrimination as well as knowledge and use of the LU [Luxembourgish] language compared to the other 2 official languages (FR [French] and DE [German])” (http://www.mipex.eu/luxembourg).

As stated above, the coexistence of inclusion and exclusion in Luxembourg can be explained by focusing on units of analysis (individuals vs. collective groups) and time horizons.

Table 1 below presents some key indicators regarding different migrant integration processes described above for various migrant categories in Luxembourg. It has compiled data presented from academic studies of migration to Luxembourg, reports from the European Migration Network’s National Contact Point and data from STATEC, Luxembourg’s statistical service.

**Table 1:** Short-term and long-term integration and exclusion of migrant groups in Luxembourg

	Short-term	Long-term
**Asylum Seekers**	88% of refugees are satisfied with their life in Luxembourg overall - housing and services are provided by the state; 68% acceptance rate of new applications from asylum seekers	70% find employment but only 50% have long-term contracts; 21% remain in precarious work; Dormitories remain physically isolated from neighboring towns which limits social interaction.
**High-skilled Migrants**	Continuous increase in numbers of residence permits issued to “salaried worker”, “European Blue Card” and “intra-corporate transferee” categories. 2018 law allows students and researchers to stay for 9 months after successfully completing studies; Salaries not commensurate with costs of living, especially amongst em-ployees of the European Union; Facilitation of socio-political integration through multilevel European citizenship	Not citizens and therefore voting exclusion; High salaries and welfare rights support families but geographic segregation imposed by elevated housing costs hinders social cohesion.
**Low-skilled Migrants**	Job market exclusion, especially due to language barriers; EU citizens enjoy employment rates that are higher than rates for Luxembourgish citizens but third country nationals have low employment rates; trade unions and NGOs; mobilize migrant integration discourse	Local voting rights and access to welfare services (over-representation of third-country nationals); inequalities in the educational system caused by social origin and the migra-tory context of pupils. Third-country nationals over-represented in Social Inclusion Income Program (REVIS); Unemployment of native citizens was 3.9% in 2017, compared to 5.8% of EU-residents and 16.5% amongst third-country nationals.
**Cross-border workers**	Represent 45% of the Luxembourgish work force; Open borders, Schengen and cross border labor market; Increase in interim contracts and labor flexibility	Lack of welfare rights which remain national; Economic integration of cross-border markets and economic inclusion

Source: Table compiled by authors based on official data (2010s)

The table indicates similar trends for all of the migrant categories in the country. Each group is characterized by short-term individual integration while long-term trends indicate collective exclusion, especially in reference to socio-economic situations. Highlighting the information in the table, we find that Luxembourg has a very high acceptance rate of asylum seekers and the country provides for their material necessities upon their arrival. However, accommodation for refugees remains isolated geographically and relatively few asylum seekers find stable employment in the country’s segmented labor market in the long-term.

Economic migrants demonstrate similar economic patterns. EMN NCP reports indicate numerous paradoxes that exist in Luxembourg’s labor market. First, the country recruits numerous high-skilled migrants to work in the financial sector and for European institutions. For this reason, there are statistically more foreigners that are employed in Luxembourg than native citizens. Moreover, most of these migrants enjoy a certain amount of political integration due to European citizenship which facilitates local voting rights and access to social services. At the same time, salaries for high-skilled migrants are not always commensurate with living costs in the country which eventually establishes serious barriers to economic integration for high-skilled migrants. Luxembourg’s National Action Plan, which was passed in 2019, has recognized access to private housing markets as a main economic challenge in the country for citizens and foreigners alike. According to the plan, this residential segregation has negatively affected social cohesion in the country. Consequently, elevated salaries do not necessarily translate into improved quality of life or social integration.

This situation is evident amongst low-skilled migrant workers as well. Official indicators from STATEC show that most third country nationals are employed in “Accommodation and food services industries,” followed by “Administrative and support service activities” and “Wholesale and retail trade; repair of motor vehicles and motorcycles” (EMN National Contact Point 2018, p. 14). As such, third country nationals are over-represented in the “at-risk of poverty” category (7.6% citizens and 24.9% foreigners) as well as unemployment rates; temporary employment rates and part-time employment. Many third country nationals work with short-term, fixed contracts. In the field of education, multilingualism has been recognized by the government as a barrier for non-Luxembourgish children who often underperform in the public school system, thus hurting their employment perspectives and social cohesion.

While these trends indicate significant exclusion, low-skilled migrants are politically integrated in Luxembourg through civil society and, as stated earlier on the macro-level, there is little open discrimination in the country. Low-skilled migrants also benefit disproportionately from unemployment insurance and social programs, which is an indicator of both political inclusion and long-term economic exclusion, thus highlighting a seeming paradox.

Finally, cross-border workers similarly, navigate the costs and benefits of the Luxembourgish labor market. On one hand, the number of cross-border workers has increased incrementally to more than 180,000 people who cross the border on a daily basis for employment purposes (Statec 2019) because salaries are comparatively high in Luxembourg and housing costs are prohibitive, forcing many to live in neighboring regions (reaping the windfall of lower cost of living just on the other side of the border). However, the system of social rights in the Greater Region of Luxembourg has penalized these workers (or prioritzed national laws and regulations). Thomas (2016) has shown how the Luxembourgish state had withheld educational subsidies for the children of cross-border workers, initially defying an order from the European Court of Justice. Clément (2013), summarizes the situation by stating that “Cross-border workers contribute fully to integration, but are not fully entitled to benefits,” thus promoting uneven development in the Greater Region. For example, Clément’s research has shown how the number of interim contracts has increased in Luxembourg in order to reduce social costs and employer obligations to cross-border workers. Clément also explains how cross-border workers have traditionally been excluded from Luxembourg’s *Tripartite*, which brings together government, employers and unions for economic planning. Burlacu and O’Donoghue (2013) have highlighted how cross-border workers can be discriminated in terms of unemployment insurance and how they pay more into this system while they work than they receive when they lose their jobs because long-term unemployment is paid from their country of residence, rather than Luxembourg.

Consequently, migrants from all of these categories contribute to and benefit from Luxembourg’s economic growth with the benefits visible at the individual level. Collectively, however, migrant groups are economically disadvantaged in the long-term, even though they participate in politcal discussions and they are active in civil society. As stated above, these seeming paradoxes are actually inherent to small state theory. This is addressed in the conclusion below.

## Conclusion

This article addresses migrant integration in small states, investigating their supposed success integrating migrants and providing stability in migration affairs. Most of the literature on small states and migrant integration focuses on the emergence of cosmopolitan citizenship and labor integration within models of economic growth. This article engages this literature through a case study of Luxembourg which is a representative small state with a long immigration history and an important migrant population representing more than 40% of inhabitants in the country.

The evidence presented in this article questions these supposed truths in two ways. Interviews conducted by the research team with government officials responsible for Luxembourg’s integration program uncovered ambivalent opinions towards migrants and migration. While officials generally supported Luxembourg’s humanitarian responsibilities, attitudes towards economic migration were mixed. In general, these officials recognized the importance of immigration for the Luxembourgish economy but they also expressed serious concern over the impact of immigration on social cohesion. These attitudes seem to contradict the literature cited above which presupposes pro-integration policy-making amongst small states.

The current situation of migrants in Luxembourg also calls into question certain conclusions in the small states immigration literature which generally focuses on economic integration presented as evidence of “successful” integration models. The empirical evidence from Luxembourg, instead presents mixed outcomes. Migrants generally enjoy economic integration on an individual level, especially in the short-term but the country’s labor market is characterized by inequality more generally. Different immigrant groups, such as asylum seekers/refugees, high-skilled and low-skilled economic migrants and cross-border workers all face important barriers to collective long-term socio-economic integration despite the presence of a strong commitment to political integration.

Through this analysis, this article directly engages with small state theory. As stated above, the characteristics of this approach, such as those identified by Katzenstein (2003) are generally accepted as positive influences on governance. According to this perspective, small states are viewed as efficient and effective policy entrepreneurs due to their supposed commitment to democratic norms and consensus-oriented policy styles. Through the above analysis of migrant integration in Luxembourg, this article questions whether small state governance characteristics are inherently positive. For example, the article has indicated through interviews with government officials that Luxembourg seems to promote solidarity (confirming small state theory) but it is not necessarily fully commited to migrant integration norms which surpass support for those in need.

Moreover, Luxembourg’s development model supports the claims that small states are open economies, they pursue niche economies and they engage in regional integration in order to enhance their international standing (in this case through promotion of the expansive Greater Region of Luxembourg). These policy choices have created wealth for the country and its inhabitants but they have also solified pre-existing socio-economic hierarchies. The economic development in the countries has entrenched bifurcated and segmented labor and housing markets.

Finally, this study questions the potential impacts of small-state consensus-building approaches to governance. Migrants seem to be politically integrated in Luxembourg’s formal institutional arrangements. However, the propensity for interpersonal relation identified by Katzenstein has contributed to political exclusion because it promotes unofficial channels of communication which accompany formal political integration processes. Migrants do not always have access to these channels to the same levels as citizens.

For these reasons, this article questions the value placed on small states in migrant integration scholarship. While integration debates may not be as vociferous or openly contentious as they are in large migrant-receiving states, small state integration models cannot be declared absolute successes. The research presented above shows how the exclusion that exists in Luxembourg is not the result of policy failures but it results from policy choices.

Where small-state theory is useful however, is in the strategies identified for addressing integration paradoxes. This article contends that small states like Luxembourg successfully promote peaceful coexistence in migration affairs by promoting a model in which integration and exclusion coexist as core characteristics that are pursued by government strategies. This derives from theories of small state governance which, as stated above, has embraced the existence of paradoxes and integrated them into policy choices. For example, language requirements (Luxembourgish, French and German) have de facto excluded (first-generation) migrants from public service employment effectively perpetuating the segmented labor market in which citizens have access to tenured public jobs and non-citzens are concentrated in the private sector in which contracts are increasingingly becoming less stable and even precarious or interim. Immigrants are simultaneously integrated and excluded which provides migrants with sufficient benefits in order to maintain peaceful coexistence but rights are differentiated enough to satisfy the privilege of citizenship (which is a comparatively “low theshold” privilege to aquire in the Grand Duchy, see: Citizenship 2020).

The glue that binds this system is Luxembourg’s welfare state. Unlike other countries where migration is perceived as a threat to welfare (see Nenghu 2020), migration is accepted as a fundamental part of Luxembourg’s welfare system (see Kerschen 2020; Amétépé and Hartmann-Hirsch 2011). The country’s commitment to welfare reflects Katzenstein’s discussion of solidarity: it goes beyond the economics of the social state as it is infused in civil society and even contributes to the perceived legitimacy of migration in Luxembourgish political debates (see Callens et al. 2012; Dennison and Dražanová 2018). 

Of course, this calls into question the sustainability of Luxembourg’s integration model because it is growth-based. The Grand Duchy has the highest population growth rate in Europe since 2000 (Statec-Atlas démographique 2020). Luxembourg is ranked first in the world in terms of nominal GDP per capita (USD 116,727 in 2020 according to Statistics Times 2020). In 2018, social spending represented 22.4% of the country’s GDP (OECD 2020) which amounts to approximately USD 15.88 billion. Can such growth and spending be sustained? Many potential obstacles exist. For example, much of Luxembourg’s wealth is generated by the Greater Region and cross-border employment. Luxembourg has successfully pushed costs of this economic integration on its partners in Belgium, France and Germany while maintaining the economic benefits which drive growth. Recently, this situation has exacerbated tensions as social movements of cross-border workers have mobilized and the European Court of Justice has ruled against Luxembourg’s claims that welfare benefits should be reserved for residents (Thomas 2016). Obviously, threats to growth, such as the Covid-19 crisis or the establishment of new economic equilibria in the Greater Region would undermine this integration model significantly. Consequently, small state “success” as a model for migrant integration, evidenced here through analysis of Luxembourg, must be questioned. Small state integration systems are based on functioning welfare states, which depend on growth in order to assure sufficient revenues to pay for services. This demographic and economic growth promotes more migration and places further pressure on social services, thus establishing a need for more growth, perpetuating a vicious cycle. Possibly the most evident paradox of small state integration systems, characterized by simultaneous inclusion and exclusion, is the fact that the more that they “succeed” in creating peaceful coexistence through segmented integration, the more pressure they generate on themselves to perform. For this reason, sustainability will the true test of migrant integration for small states as they further integrate into global economies.

## Data Availability

Statistical data utilized in this article is publicly available from third party sources that are cited in the text. Interview datasets generated and/or analysed during the current study are not publicly available in order to protect the anonymity of interviewees who participated in the study. Anonymized data is available from the corresponding author on reasonable request. The unpublished Evaluation PAN-ILD (2013) was released for academic research/use by Gary Kneip (President of the CES, 2013–2015).
